# Crafting urban equality through grassroots critical pedagogies: weave, *sentipensar*, mobilize, reverberate, emancipate

**DOI:** 10.1177/09562478221115334

**Published:** 2022-09-05

**Authors:** Adriana Allen, Julia Wesely, Paola Blanes, Florencia Brandolini, Mariana Enet, Rodrigo Faria G Iacovini, Rosario Fassina, Bahiá Flores Pacheco, Graciela Medina, Alejandro Muniz, Soledad Pérez, Silsa Pineda, Marilyn Reina, Luz Amparo Sánchez Medina, Juan Xavier

**Keywords:** critical pedagogy, Latin America, pedagogic practices, popular urbanism, urban equality

## Abstract

How do ordinary citizens, activists and urban practitioners learn to become agents of change for a socially just habitat? The paper explores this question through the experiences of eight grassroots schools of popular urbanism working under the umbrella of the Habitat International Coalition (HIC) in Latin America. Building on a process of self-documentation and collective pedagogic reflection driven by the protagonists of these schools, the analysis explores the core pedagogic practices identified across the schools to enact popular urbanism as a collective and intentional praxis: to weave, *sentipensar*, mobilize, reverberate and emancipate. We argue that, put in motion, these pedagogic practices transgress the rules and boundaries of the formal classroom, taking participants to and through other sites and modes of learning that host significant potential to stimulate collectivizing and alternative ways of seeking change towards urban equality.

**Figure:**
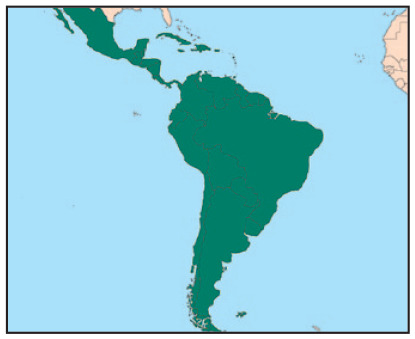


## I. Introduction

*The academy is not paradise. But learning is a place where paradise can be created. … This is education as the practice of freedom*.^(1)^

In this quote, bell hooks invites us to discover the practice of freedom through learning; not as a practice of individual freedom but as a collective way to transform the world we live in. In this paper, we look at sites of learning other than the academy, where paradise can be created. We reflect collectively on the pedagogic practices of eight schools of popular urbanism working under the umbrella of the Habitat International Coalition (HIC) across Latin America, and on their capacity to activate critical agency to confront urban inequalities and advance habitat-related human rights.

HIC is a global alliance currently constituted by over 350 member organizations (of which about a third are based in Latin America), which struggle collectively for greater social justice, gender equality and environmental sustainability. For over 45 years, the Coalition has brought together social movements, non-governmental organizations, community-based organizations and activist and research institutions with a common political purpose: mobilizing their collective muscle from local to global scales to make visible, defend and produce habitat rights.^(2)^ In keeping with its collective orientation, the Coalition’s structures (its Board, General Secretariat and Regional Coordination Offices) follow the mandate of HIC’s Members, who are their governing body.

The schools analysed here are not one institution, but rather an assemblage of diverse pedagogic experiences. They are run by HIC members, friends and allies in Latin America, and each school has a high degree of autonomy. Their pedagogic practices are encapsulated in formative processes: in the building of cooperative housing movements in Uruguay, and the capacity-building of Colombian taxi driver associations to become inclusive in their work with people with disabilities; in the learning encounters between Indigenous populations and low-income communities in San Martín de los Andes in Argentina, and the reclaiming of bodies and public spaces from a feminist perspective in Córdoba, Argentina, to name a few. Despite their diversity, the schools have in common the political parity of learners and pedagogues (who include community leaders, cooperative members, youth, women’s groups and local government officials, among others), as well as a commitment to horizontal, counter-hegemonic learning to act in advancing struggles through popular urbanism.^(3)^

Most of the schools analysed here are part of the HIC Latin America (HIC-AL) working group on the social production of habitat. This group encompasses a great diversity of theoretical approaches, from those rooted in a Marxist perspective that ultimately seek to restore the social function of land and housing, to feminist and decolonial perspectives rooted in the assumption that theory, knowledge and practice are constantly re-made through activism and resistance. In this sense, the Coalition has acted over the years as a resonance chamber, enabling member organizations to exchange and mature their practices in critical dialogue with others.^(4)^ In a similar vein, HIC-AL’s involvement with the schools is expressed in several ways, from providing inputs into their pedagogies, concepts and content, to nurturing and expanding their regional networking capacity. Since 2003, HIC-AL has also hosted a working group on capacity-strengthening which has spearheaded the consolidation of various pedagogic experiences from its members and articulated some of them into formal education programmes (including the Diploma Course in Participatory Design, one of the experiences analysed in this paper).

**Figure 1:**
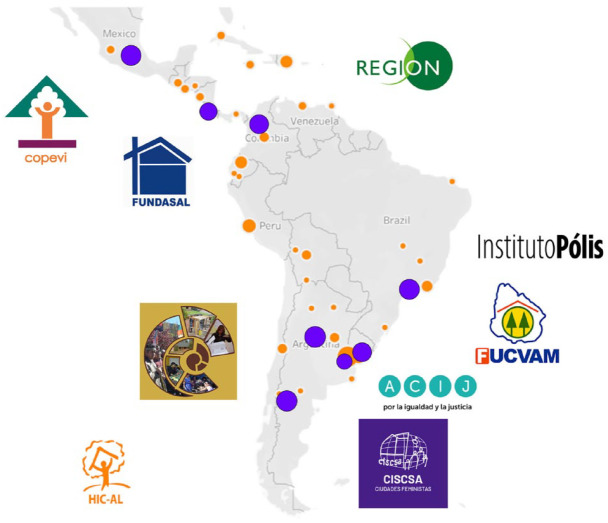
Location of HIC-AL members (orange dots) and the eight schools (violet dots) analysed in this paper SOURCE: Authors.

How do these diverse pedagogic practices contribute to achieving their transformative and transgressive vocation? Or, in other words: How do participants learn to become agents of change for a socially just habitat? To respond to these questions, in June 2019, the two first-named authors of this paper (one of whom became president of HIC in 2020), embarked on an in-depth pedagogic exploration of the schools under the HIC-AL umbrella as part of the capacity-building and action research programme, Knowledge in Action for Urban Equality (KNOW). The research initially involved 21 extensive conversations with 14 HIC-AL members and affiliates, online and in person in Argentina, Colombia, Chile, Cuba and Mexico.^(5)^ As the COVID-19 pandemic brought the possibility of further face-to-face encounters to a halt, we shifted to a multimodal way of working, which included a process of self-documentation and analysis by schools interested in this reflexive^(6)^ exercise and collective online workshops devoted to identifying the core “pedagogic narrative” underpinning each school, as well as their common practices (see Figure 1).

The remote phase of engagement evolved around the production of short films by each school and a collective documentary, as entry points to distil the core and common pedagogic practices that have coalesced under the umbrella of HIC-AL. In this context, the short films and documentary acted as catalysts for horizontal conversations on the role of popular urbanism pedagogies in nurturing struggles for habitat rights; that is, articulating critical and political learning processes as pathways towards strategic change to address inequalities. Supported by La Sandía Digital – a feminist collective of filmmakers based in Mexico – this horizontal process involved four online workshops with around 15 participants each, between August and November 2021, in which pedagogues and communication teams from each school developed their story from a pedagogic viewpoint. These exchanges evolved from oral accounts to audiovisual narratives and culminated in a fifth comparative analysis workshop through which all participants identified the practices that make their pedagogic approaches critical and counter-hegemonic. Between the workshops, the pedagogic reflexion involved a larger number of participants, as each school engaged in searches through their own archives, focus group discussions, one-to-one interviews, storyboarding and filming.

The following section outlines key contributions and principles of critical pedagogy within the field of popular urbanism in Latin America. We then analyse the five core pedagogic practices collectively identified by the eight schools: to weave, *sentipensar* (feel-think), mobilize, reverberate and emancipate.

## II. The Role of Critical Pedagogy in Popular Struggles for Habitat Rights

Building on historical experiences, such as the *Hacedores de Ciudad* in Venezuela and large-scale movements of *autogestión* in Peru in the 1960s, numerous authors have highlighted the impactful scale and scope of popular urbanization in the context of Latin America. The strategy is recognized as a set of practices and processes through which the hegemonic logics of the market, political regulation and fiscalization in issues of land, housing and territory are contested through bottom-up, citizen-led actions.^(7)^

While this body of research often refers to “popular urbanization” and “popular urbanism” as interchangeable notions, we argue for recognizing their difference. Popular urbanization pertains to the processes by which low-income and marginalized women and men typically produce and appropriate the urban territory. In contrast, popular urbanism – the arena in which the schools thrive and the focus of the present paper – refers to a domain of practice that contests the institutional power of urbanism as a discipline, and the legitimacy it enjoys in producing knowledge and practices. Thus, popular urbanism encapsulates a collective intentionality to reclaim the authority and legitimacy of other ways of producing and using the city as a commons; the ability to craft a different relationship with state institutions across periods of dictatorship and (re)democratization^(8)^ and conscious and explicit efforts to valorize the experiences, pedagogies and principles that have redressed social and environmental inequalities and injustices over time, and that continue to do so.

Within Latin America, historiographic work has highlighted the political and socio-spatial complexity of popular urbanism throughout the 20th and 21st centuries.^(9)^ Tracing the genealogy of popular urbanism across Latin America has been a central project for many regional activists and academics over the years, with a notable example being the work of the architect Enrique Ortíz Flores, a member of the non-governmental organization COPEVI since 1965, and one of the co-founders of HIC in 1976.^(10)^

To talk about popular urbanism is thus to recognize other ways of making the city, parallel to the rules established by the institutions of government and academia, and to the logics of capital.^(11)^ In this conception, popular urbanism hosts substantial potential to redress prevalent urban inequalities in four key dimensions: first, by working towards the fair redistribution and commoning of the goods, services and opportunities that are integral to the realization of habitat-related rights; second, by amplifying the voices and claiming recognition for differentiated identities and knowledges, particularly of those engaged in pushing the boundaries for urban equality on an everyday basis; third, by advocating for their parity in political participation, not just in invited spaces, but through claimed spaces of city-making; and last, but not least, by fostering urban practices built on mutual care and solidarity, as well as on state responsibility.^(12)^

Thus, popular urbanism is a conscious political practice; a practice identified with by social movements, NGOs, research institutions and other supporting organizations from the eight schools participating in this collective reflexion, as well as many others across the region. Although not all these pedagogic experiences call themselves “schools”, many self-identify as such in recognition of their alignment with the foundational work of Paulo Freire.^(13)^ It is the practice of critical pedagogy, with its ethical and political intentions, that is key to re-signifying where popular urbanism learning happens and how it works. As critiqued by Giroux,^(14)^ the school, as the main social instrument and space devoted to education, has become over time an instrument of students’ social assimilation rather than a space to engage critically with society and strengthen capacities to change it. Adhering to this critique, the work of the schools discussed in this paper encapsulate a conscious effort towards reclaiming the emancipatory power of popular education.

Although Freirean critical pedagogy has its roots in rural peasant movements in Brazil, it has long been embraced by, and expanded into, urban realities as well as more intersectional and feminist perspectives on popular struggles. Black, Indigenous and feminist scholars, in this context, have brought forward notions of the oppressed in relation to structural racism and patriarchal domination.^(15)^ Freire’s foundational work^(16)^ in critical pedagogy emphasized the dialogical relation between theory and praxis, as well as the importance of giving this praxis a direction towards change for social justice, nurtured by iterative and deep processes of reflexion. His conceptualization of critical pedagogy as freedom through learning benefited over the years from contributions from postmodern, feminist, postcolonial and queer theories and, perhaps most importantly, has continued to be a central practice of social movements and organized civil society.

According to Sara Motta, the critical pedagogies underpinning popular urbanism do not aim to create an *“alternative monological practice of life”*, but rather aim for the *“pedagogical [to] become an essential part of creating the openings, possibilities and relationships to enable communities that are often silenced and violently invisibilised to appear as embodied political subjects”*.^(17)^ To achieve this end, learning through, with and in struggle, requires a pedagogy that transgresses the rules and boundaries of the classroom as a site of learning and *“instead embed[s] educational practice in multiple spaces: the community, the workplace, the co-operative, the home, the self”*.^(18)^ Moreover, the content of learning is co-constructed rather than being based on predefined curricula, which is responsive to context-specific urban knowledges of learner-pedagogues as well as to the inequalities and injustices the learning process seeks to confront. As argued by Motta, for learning to become emancipatory, pedagogical practice needs to be the *“product of praxis at the collective level of lived experience”*,^(19)^ in which counter-hegemonic knowledges and (hi)stories of resistance and hope are cultivated.

While these counter-hegemonic knowledges and ways of knowing (*saberes*) have received attention in discussions on critical pedagogy and popular urbanism,^(20)^ relatively little attention has been given to the pedagogic practices (*haceres*) that nurture and sustain them. Thus, this paper centres on *how* learning activates collective agency for a socially just habitat, aiming to elicit pedagogic practices that stimulate alternative ways of seeing, listening, being and seeking change.

## III. The Schools at Work: *Haceres* and *Saberes* as Pedagogic Practices

Table 1 provides an overview of the eight schools participating in this pedagogic reflexion, and their convenors, learners, purpose and pedagogic approach.^(21)^

**Table 1:** Description of the eight pedagogic experiences documented and analysed in this paper

Name and location	Convenors	Learners	Aims	Pedagogic approach
*Diplomado Territorio y Hábitat –* Diploma on Territory and Habitat; Mexico City	Universidad Autónoma de la Ciudad de México, Movimiento Urbano Popular, Centro Operacional de Vivienda y Poblamiento, AC (COPEVI)	Members of collective and neighbourhood organizations, Indigenous inhabitants of the city and university students	To develop critical, reflexive and propositional thinking; based on popular urban movements’ perspective on *buen vivir*, participatory democracy and territory and habitat rights.	Focusing on dialogic methods, inter-learning and the co-production of knowledge among participants, the Diploma is structured across 4 online modules over a period of almost 8 months. Before the pandemic, the course also included field visits.
Cooperativism and women-led housing construction and habitat processes; Potrerillos and La Palma, San Salvador	Fundación Salvadoreña de Desarrollo y Vivienda Mínima (FUNDASAL)	(Female) community and cooperative members.	To strengthen women’s knowledge and capacities to claim and enact their habitat rights.	From self-recognizing one’s rights to constructing them collectively through women-led housing, FUNDASAL’s pedagogy works through a mix of workshops aimed at developing a new consciousness and hands-on sessions on self-construction techniques.
*Medellín Ciudad Accesible* – Medellín Accessible City; Medellín, Colombia	Corporación Región + Fuerza Incluyente	Neighbourhood organizations, collectives of disabled people, and advocacy target audiences (such as taxi drivers, local government officials and the general public).	To strengthen participants’ consciousness and capacities to advocate an intersectional perspective in building mobility as part of the Right to the City.	Largely experiential pedagogies, which engage with and through the realities of differently-abled people, for example, through discussions, events in public squares, shared walks and transits through the city.
Cooperativism schools, with a particular focus on intergenerational and gendered learning and relationships with the state; across Uruguay	Federación Uruguaya de Cooperativas de Vivienda por Ayuda Mutua (FUCVAM)	Current and future members of cooperatives, with some learning processes particularly focused on youth and women.	To nurture, sustain and mobilize the housing cooperative movement as a key strategy to reclaim the social function of housing and land.	Capacity-building is integral to the working of cooperatives and operates through different modes of learning, including learning-by-living/doing, decentralized workshops, and peer learning across cooperatives.
Feminism schools – *Voces de Mujeres Diversas por Ciudades Seguras, Inclusivas y Sostenibles* – Voices of Diverse Women for Safe, Inclusive and Sustainable Cities; Córdoba, Argentina	CISCSA Ciudades Feministas	Community leaders and members of women and feminist organizations, as well as women working in urbanism and urban planning.	To strengthen debates on the rights of women and diversities to the city; to build social force around the experiences and demands of those identifying as women, trans and lesbians; to develop concrete proposals that advocate for a feminist city.	Capacity-building and participatory workshops developed through feminist methodologies, such as *“cuerpo-territorio”*, visioning exercises, manifestos, public manifestations and celebrations.
*Diplomado Iberoamericano Diseño Participativo Sustentable del Habitat –* Ibero-American Diploma in Participatory Sustainable Habitat Design; LA Regional	Universidad Nacional Autónoma de México in collaboration with HIC-AL and Taller 36 of the National University of Cordoba	Anyone interested in the social production of habitat, including leaders of grassroots organizations, university students and professionals of different disciplines.	To learn and apply concepts, methodologies and tools for the social production and management of habitat, which are not taught in regular design courses.	Inter-learning spaces as well as group and individual work across 210 hours, 2 x 2 hours of live sessions per week (workshops) and 8 hours of independent learning.
*Escola da Cidadania* – Citizenship School, with a cycle of 16 different thematic courses (such as culture and cities, climate justice and urban infrastructures, etc); São Paulo, with online participants across Brazil	Instituto Pólis	400–500 online learners with diverse identities and backgrounds (in terms of professional experience, gender, age, geography and others). Core target groups are social movements and public officials.	To be a space for reflection and formation of critical thinking in the different dimensions of constructing citizenship: human rights, right to the city, activism and public policies.	Polis Institute applies creative pedagogic practices, such as the collective development of thematic playlists, and the use of social media as a means to extend the learning beyond the boundaries of the school. Practically, each thematic course consists of six online 2-hour weekly sessions, with voluntary discussion groups in between sessions.
*Programa de Formación en Derechos para Referentes Barriales* – Formative Programme in Rights for Community Leaders; Buenos Aires 2019, now online and with reach across Argentina	Asociación Civil por la Igualdad y la Justicia (ACIJ)	In 2019 ACIJ’s school brought together 60 community leaders, including representatives of social and political organisations in informal settlements, and independent leaders, who seek to incorporate or reinforce within their repertoire of public actions, the use of legal tools and the language of rights as a way to strengthen their interaction with public authorities.	To promote communities’ understanding of their rights as guaranteed by the legal system. To strengthen grassroots organizations’ capacities to claim their right to the city and to claim parity of political participation in their neighbourhood improvement and redevelopment processes.	ACIJ’s approach is learner-centred and works through horizontal exchanges that valorize the knowledges and practices of community leaders as pedagogues. The curriculum is therefore open and flexible to respond to specific needs, contexts and challenges and typically works through 13 sessions/4 modules/40 hours of classes over three months. Sessions are facilitated as interactive workshops – with space for debates and role plays.

SOURCE: Authors.

We now turn to the five verbs that encapsulate the core pedagogic practices identified by the participating schools as crucial means to foster the articulation of *saberes* and *haceres* underpinning popular urbanism. While not all these verbs are present equally across all schools, they resonated strongly with every participant, as critical practices with profound potential for activating and sustaining change. We explore each verb starting with a vignette from a sequence of the films produced by the schools, followed by a discussion of what each critical pedagogic practice involves in developing key capacities to tackle urban inequality.

### a. Weave

On a sunny day, an architect and an Indigenous inhabitant of *Barrio Intercultural* stand on an empty lot, holding in their hands a model of one of the planned buildings in the neighbourhood. As their hands move around the model, the light creates shadows that get them to imagine together how the building might be used at different times of the day and what that would mean for different collective uses and members of the community.

In the *Barrio Intercultural* (Intercultural Neighbourhood) in the south of Argentina, inter-learning spaces bring together diverse professions, social identities, knowledges and practices in dialogue with each other. Inter-learning, in this context, refers to a process whereby the intentional juxtaposition of different, contrasting and complementary knowledges and practices generates new forms of living and working together. This school emerged from an alliance between *Vecinos Sin Techo* (Neighbours without a Roof) and the Curruhuinca *lof* (community) of the original Mapuche people in San Martín de los Andes.^(22)^ Initially unified by their common need for housing, over time the development of their Intercultural Neighbourhood became a means to coexist in a common place rooted in the cosmovision (or worldview) of *“buen vivir”*. As argued by Eduardo Gudynas, this cosmovision embodies community-centric, ecologically balanced and culturally sensitive conviviality.^(23)^ It encapsulates perspectives that are a far cry from market-led approaches to housing, land and services and seeks to build new forms of public coexistence, in diversity and in harmony with nature. In this experience and across those of other schools, weaving is the key pedagogic practice that crafts a new material and social fabric by interlacing threads of collective dreams, rights and aspirations.

Weaving is not just about connecting different knowledges and actors, but a process of rooting contemporary struggles in a rich historic trajectory of learning from, for and in political struggle. In the case of the school of *Barrio Intercultural*, contemporary collective dwelling practices were reimagined with the Mapuche people – and thus re-embedded in their ancestral forms of being part of the territory. Here, the school has been the open space that connected historical and contemporary demands and ways of being in dialogue with Western critiques of capitalism, particularly from the field of feminist thought and environmentalism. This dialogue not only wove together narratives and experiences of oppression but also alternative ways to dwell in the territory as an intercultural community, which over time opened new institutional possibilities for the recognition of social production of habitat processes.

The practice of weaving together historically rooted and contemporary habitat struggles is also present in the pedagogic practices of the Ibero-American Diploma in Participatory Sustainable Habitat Design, a regional experience with 22 organizations that actively engages with the *Barrio Intercultural* in its content and pedagogy. The diploma also draws from the pedagogical practices of the so-called *Taller Total* in the early 1970s, a model in which architecture was understood as a social practice, and in which “users” or dwellers play the same role as trained professionals.^(24)^

*Taller Total* set a radical precedent across the region, aiming to open up the university and transform the education of urban practitioners through a collective transdisciplinary pedagogical experience. Originally implemented at the National University of Córdoba (Argentina), over time the pedagogic principles and practices of *Taller Total* were adopted in other locations and disciplines. While this wave of radical pedagogies was brought to a halt in 1975 by the repressive dictatorship of 1976–1983, its legacy continues to be re-lived by contemporary experiences such as the *Barrio Intercultural* and the diploma course. In these experiences, the key tenets of *Taller Total* are reactivated under contemporary circumstances, prompting a dissolution of disciplinary boundaries converging in the “habitat” problematic by building a critical history of the habitat and its social production, while weaving in new capacities and possibilities for social transformation.

Hence, weaving as a historical and relational pedagogy generates an inter-learning space that enables the interaction between different *saberes* and *haceres*, rooting them, problematizing them, questioning their assumptions and generating new ways to frame a given problematic. Unlike conventional pedagogies, the capacity to act does not rest on having full knowledge and control of the problem and the solution, but rather on the capacity to engage with uncertainty, singularity and conflict, and to recognize and deploy the social production of habitat as long and open-ended processes of dwelling that will continue to change over time.

### b. *Sentipensar*

In a community centre in Córdoba, Fada lies on the floor, the outline of her body being drawn on a big sheet of paper. *“Now, let’s think where we locate our emotions, which form they have, and where we want to put them on our drawing of the body”*.^(25)^ Paola guides the group through an exercise of *cuerpo-territorio*, a feminist methodology often used in the context of violence and inequalities to reflect on the body as *“means to diagnose territorial conflicts and to initiate healing of bodies and territory”*.^(26)^

*Sentipensar* can be roughly translated in English as the capacity to “feel-think”. *Sentipensar* refers to pedagogies that cultivate sensibilities and affections that see the human and non-human world as interdependent and in constant flow. It invites us to engage with the energy that flows and interacts between situated minds-bodies and action in their full diversity and highlights the role of the medium and the media in the construction of *haceres* and *saberes*.^(27)^

*Sentipensar* offers a transformative pedagogic practice that emphasizes the complementary relationship *“between the* ***sentir*** *of intuition and the inner life and the* ***pensar*** *of intellectualism, between tacit knowledge and wisdom; between Western and non-Western ways of knowing and doing.”*^(28)^ Practised across several of the schools as a way to learn and as a learnt capacity, it departs from our language and affections as sites that actively build the world in which we are immersed in its full complexity. It challenges the Cartesian separation between us humans and nature, as well as the separation between us and others. From this perspective, to co-learn is to develop simultaneous capacities to be, to know, to do and to care.

The potential of *sentipensar* to confront urban inequalities is multiple and most deeply realized through the way it advances epistemic justice – not just by including typically marginalized *saberes* but, more deeply, by awakening new ways of making sense of the world from our senses, affections and positionalities. This critical pedagogy practice adopts feminist and decolonial thought, insisting on the plurality of knowledges, to acknowledge the diverse points of departure and inequalities that, as argued by De Jong and colleagues: *“emerge from the intersections of race/ethnicity, class, gender, sexual orientation, age, body ableness and so on”*.^(29)^ This pedagogic practice further emphasizes the importance of situating the present in historical perspective in order to unveil the roots of different forms of oppression.

**Figure 2:**
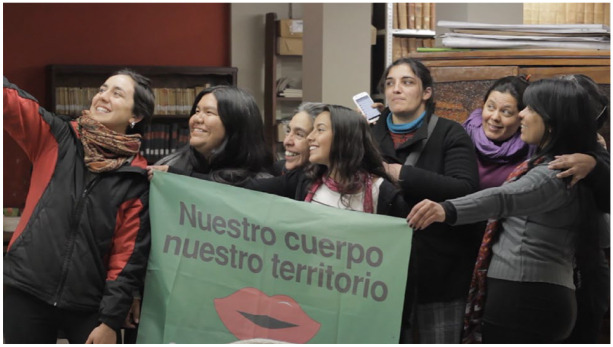
“Our body, our territory” SOURCE: CISCSA et al. (2021).

In the above vignette, CISCSA’s feminist school works from the scale of the body to understand the household, the neighbourhood, the city and the wider territory (see Figure 2). This engagement with felt and sensed areas of pain and joy, of repression and freedom, allows learners to connect with their own maps of *sentipensar* and those of others, and to trace patriarchal and racist genealogies, active in the present way in which women’s bodies can and cannot inhabit the city; aiming, in short, to counteract their disembodiment from the city.

Like weaving, this approach resonates with Ivan Illich’s work on learning webs,^(30)^ through which he argues that learning requires unlearning the ways in which our bodies and minds have been schooled and tamed. The un-taming of the schools under the HIC umbrella in Latin America through *sentipensar* practices should not be confused with the development of empathy, but rather with radical ways to see the world, to break with deeply learnt forms of oppression and control that inadvertently travel from our bodies into our homes, neighbourhoods, public spaces and cities. Here, *sentipensar* practices activate new forms of caring and conviviality. This approach is also clearly expressed in the *Barrio Intercultural*, where the neighbourhood is conceived, built and lived as a place for mutuality and conviviality.

### c. Mobilize

**Figure 3:**
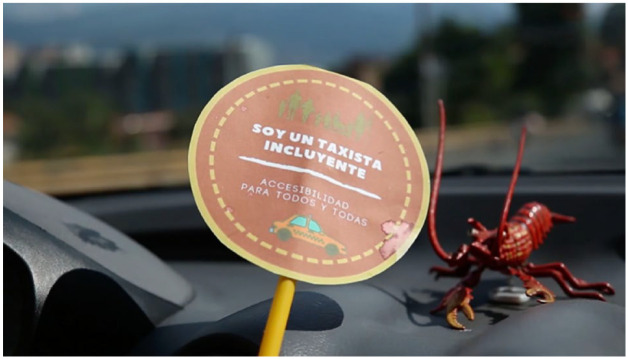
“I am an inclusive taxi driver” SOURCE: Corporación Región et al. (2021).

In the hillsides of Medellín, members of *Fuerza Incluyente*, a collective of people with disabilities, wait for a taxi to get to the city centre. As a taxi driver stops, the group engages in a conversation with him, first showing him how to dismantle and store a wheelchair in the taxi boot. Then, Nancy – who has short upper limbs – takes a seat and instructs the taxi driver to put her seatbelt on. On their way, the conversation covers a range of topics – from Nancy sharing her experience with verbal abuse, to the ways in which people with disabilities can be addressed in a dignified manner. At the end of their ride, she hands the taxi driver a sticker to put behind his windshield (see Figure 3). It declares him an “inclusive taxi driver”, that is, one of many transport providers across the city who have taken part in a pedagogic experience of learning from people with disabilities and who commit to practising inclusive transport services.^(31)^

In this vignette, the taxi becomes the classroom, the co-learning space where women and men with different disabilities share their experience of the city with taxi drivers and, in the process, build new ways of understanding how their bodies interact in a common territory. The pedagogic practice of “mobilizing”, in a literal sense, fosters the inclusion of marginalized groups in the city, not merely by adapting the city to their particular needs through retrofitted urban design, but by advocating for more pluralistic and intersectional perspectives on urban mobilities. In this school, *Corporación Región*, working together with *Fuerza Incluyente*, co-developed an experiential pedagogy which opens spaces to encounter diversities, enhancing possibilities for people with disabilities to move around the city, while simultaneously generating a new consciousness among key actors and institutions responsible for enhancing mobility for all.

Another way in which these mobilizing pedagogies address inequalities is through the strategic role that education assumes in social movements for facilitating the transfer and expansion of knowledges over generations, as well as across territories. In the Uruguayan Federation of Mutual Aid Housing Cooperatives (FUCVAM), intergenerational learning has been a strong pillar of its work as a movement. This, on the one hand, concerns the everyday learning through which young people living in cooperatives acquire values of mutual care and solidarity, as well as consciousness of the wider political project within which they are growing up. As Ignacio Lostorto, a young adult in FUCVAM’s cooperative school states: *“[mutual care] is the most natural, beautiful, and productive way to advance, and it is the way that promotes values such as conviviality and which gives you tranquillity with yourself and with others”*.^(32)^

On the other hand, FUCVAM has evolved and systematized the promotion of intergenerational learning through modular pedagogic systems that can be adapted to specific needs and challenges among cooperative members. For instance, confronted with the challenge that young adults often left the cooperatives after moving out of their parents’ house, the Federation started the so-called *“nucleo de promoción juvenil”* (youth educators programme). Javier Vidal, the former coordinator of formative activities at FUCVAM, highlights that this initiative: *“did not intend to fill the physical premises of the Federation with hundreds of young people, but rather aimed to work in a decentralized manner across the territory, so that young people can build their own spaces in the cooperatives and, in turn, create new cooperatives when they move out, when they socialize, when they start their families. That worked pretty well, we had many cooperatives originating from this”*.^(33)^

Thus, mobilizing pedagogies operate both as a means to enhance everyday mobility from an intersectional perspective and to promote intergenerational learning and mobilization within social movements. To mobilize involves seeking change through moving literally across the city as well as by mobilizing collective agency. Through these practices, mobilizing becomes a means to common the city, and to restore its social function and production, opening the space for sustained and expanded action.

### d. Reverberate

Flavia has not missed a single session of Polis’s Citizenship School. Since the start of the pandemic, she felt isolated in her struggles for the right to the city, and overwhelmed by the political neglect with which they are ignored. Fighting isolation, she has become active on social media platforms, where she engages in discussions initiated by the school that resonate with her. In the process and inadvertently, she is expanding the learning process beyond the school, allowing her “aha” moments and reflections to reverberate with a far wider community.

**Figure 4:**
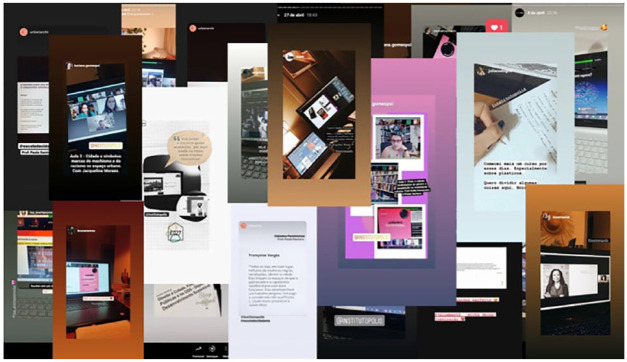
Snapshots of reverberations of the School of Citizenship on Instagram SOURCE: Authors’ personal communication with Polis Institute based on raw footage shared by them.

Following its relaunch at the beginning of the pandemic, Polis Institute’s Citizenship School in Brazil provoked unanticipated pathways and practices of learning, as its pedagogies started to reverberate from curated online sessions to social media channels like Instagram (see Figure 4). There, stories and observations from the virtual classroom were shared and further debated, while new conversations led to thousands of new followers. Engagement with the activating effects of social media and their political power, for example, through proactive engagement with “influencers”, has since become a strategic leveraging practice for the pedagogues running the school.

The notion of “reverberation” is widely applied in sound studies,^(34)^ not only with a physical but also a political meaning. In this context, to reverberate means the putting-in-motion of resonance, to amplify diversity perspectives, to grant them a dignity of attention and recognize their value and importance. As a pedagogic practice, reverberating is led by learners as they synthesize their own sense-making of how, for example, a transgender person experiences their right to the city, while provoking further reflections that together build new collective understandings, creating social proximity even in a context of physical distance. As such, to reverberate sets off new possibilities to act across spaces, temporalities and struggles that go beyond those curated by the schools.

Moreover, reverberation provokes, in Gershon’s words, *“a collapsing of distance across spacetimes so that the reverberations of resonances come crashing down on individuals, groups”*.^(35)^ Hence, reverberating pedagogies also give rise to the travelling of ideas and practices across the region through relational and translocal learning. For instance, in the online Citizenship School of Polis, one participant from northern Brazil managed to successfully fight an imminent eviction with the tools and capacities acquired from a participant from another region, which resonated with him. In a similar way, the participatory design process undertaken in the *Barrio Intercultural* in Argentina generated reverberations through a shared framing of the right to housing as a collective practice embedded in conviviality with one another and with the territory. Moreover, the process of “weaving” pedagogies described before, here reverberates to a large number of learners and organizations, leading to the formation of new collectives working on participatory design in the social production and management of habitat.

### e. Emancipate

**Figure 5:**
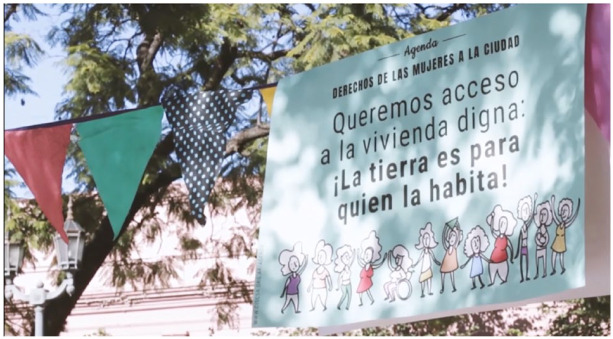
“We want access to dignified housing: The land is for those who inhabit it!” SOURCE: CISCSA et al. (2021).

Amidst laughter and the creation of an unfolding collective manifesto towards a feminist city, Paola reflects on CISCSA’s feminist school: *“Something that we seek all the time in this work process is to encourage ourselves to imagine other possible realities that we always had on the horizon. We conduct several exercises to be able to make room to imagine a feminist city in which we would like to live [Figure 5]. This means to give rise to the power of the feminist imagination, to the possibility of imagining and dwelling cities from other more beautiful, fairer, more habitable, more loving places.”*^(36)^

Across all the schools, pedagogy is critical to the project of emancipation, and emancipation critical to urban equality. As a pedagogy, to emancipate does not just evoke a destination but a journey; a journey through which the capacities to dare and to imagine a different city are activated. This intention counteracts the pursuit of liberal and andragogical theories of learning and brings to the fore the development of a critical consciousness that exposes and confronts the oppressive structures which confine and limit our experience of the urban and of the right to the city.

Drawing on Thompson’s work,^(37)^ while raising the case for critical pedagogy in nursing education, Jane Harden argues that *“everyday living as experienced by the majority is characterised by a naive, pre-reflective adherence to ‘established’ versions of the life world. The consequence of this is that ‘factual’ or doxic patterns of living are never challenged, and the question of legitimacy is never raised, because the social world is presented and accepted as a natural phenomenon”*.^(38)^ In this sense, a critical parallel can be established between nursing and urban planning education. For a long time, professional education, in this and other fields, has hampered critical awareness and responses from learners, inadvertently turning professions of care into professions of oppression. In the case of nursing education, the dismissal of emancipatory capacities has indeed turned it into a site of oppression for those practising the profession, typically women, who are subordinated to a subservient role.

Critical pedagogy is fundamentally about hope, liberation and equality;^(39)^ it is about *conscientisation* – that is, the process of developing a critical awareness of one’s social reality through reflexion and action – and about counter-hegemonic practices, which are the backbone of emancipatory pedagogies in the full sense of Freire’s educational practice theory. To emancipate thus implies to transform the internal conflict experienced by subordinated or colonized groups in an oppressive society, a conflict defined as *“double consciousness”* by W E B Du Bois.^(40)^ This calls for activating a shift from seeing ourselves simultaneously through our own eyes and from the perspective of oppressive systems such as racism, patriarchy, colonialism or neoliberalism to rediscovering and asserting our social identities as crucial sites to imagine more socially just visions for the future of the city.

Activating a new consciousness – one that leads to the very possibility of enacting counter-hegemonic and transgressive visions and practices of inhabiting a more equal city – is at the core of the schools working under the umbrella of HIC-AL. In the school driven by *Corporación Región* and *Fuerza Incluyente* in Medellín, this translates into the ways in which an inclusive city is approached: not as one that accommodates the needs and experiences of people with disabilities through marginal artefacts and ad hoc interventions such as ramps, but rather by turning accessibility and mobility for all as a default position from which the city is planned and managed. In a similar vein, FUCVAM’s schools of cooperativism do not build houses to be traded as assets in the real estate market, but as homes to be inhabited, as concrete political practices that reclaim the social function of housing and land in cities – where, as expressed in the title of their short film, *“the dwelling is not the end, it is the beginning”*.

CISCSA’s feminist school explicitly employs emancipatory pedagogies that valorize situated experiences as opportunities to reclaim the city, and align with Sara Motta’s call to *“deconstruct subordinated and naturalised social and spatial relations, while looking at gender, race, age, class, sexual orientation and mental and physical ability as one”*.^(41)^ These emancipatory pedagogic practices work through power and consciousness-raising, acknowledging the existence of oppression, as well as the possibility of ending it, and foregrounding the desire and capacity for social transformation. These practices manifest through the use of open – non-predetermined – learning strategies that encourage participants to share in a safe space their own intersectional positionalities and experiences with those of others through written, spoken and visual reflections.

## V. Creating Paradise Beyond the Classroom

To weave, to *sentipensar*, to mobilize, to reverberate and to emancipate – these verbs together articulate the grammar of the schools coalescing under HIC in Latin America. The power of these actions should not be underestimated, as together they convey the ways by which critical grassroots pedagogies can activate collective agency and capacities to carve pathways towards urban equality.

Teresa Caldeira^(42)^ contends that as ordinary citizens engage in making the urban, they often become fluent in claiming their right to the city. Working through the critical pedagogies examined in this paper, we see that such fluency is not acquired spontaneously or individually, but as the outcome of collective learning processes that turn habitat struggles into sites of transformative change by linking learning with mobilization, advocacy and action.

As argued by Paulo Freire,^(43)^ the main method of critical pedagogy is dialogue, a dialogue adapted to each context, in which everyone can actively participate through the following process: (a) by encouraging women and men to develop their own critical consciousness in order to effect change in their world through social critique and political action; (b) by critically understanding one’s own practice; and (c) by changing our practices in order to tackle common struggles and act upon reality. By collectivizing consciousness, resistance and contestation, as well as possibilities, sensibilities and hope, we are witnessing a contemporary reinvention of popular urbanism, driven by social movements, as a simultaneous practice of what Motta and Esteves call *“pedagogising the political and politicising the pedagogical”*.^(44)^ This double practice challenges the logics of neoliberal capitalism, of patriarchy and of racism and their hegemonic translation into systemic urban inequalities. It does so by subverting the marginalization of ordinary citizens, by embodying learning in their practices and in the everyday life experiences of the urban, by generating spaces to unlearn the oppressor’s logic, by re-signifying the social and the public, and by pluralizing the *saberes* and *haceres* that make a just city a concrete field of imagination and action.

The five pedagogic practices shape the four dimensions of urban inequality as well as the links between them. Emancipatory pedagogies, for example, have been fundamental in making visible, revalorizing and seeking a social redistribution of the roles of women as carers, particularly pertinent in pandemic times. The experiential pedagogies in Medellín were critical for people with physical disabilities to break away from their isolation in the hillsides of the city, towards actualizing their rights and opportunities in the city. In terms of reciprocal recognition, the practices of *sentipensar* in particular provoke profound reflections not only on the question of what and whose knowledge counts in habitat struggles, but how relations between different knowledges, feelings and practices – professional, embodied, experiential and others – are constructed and enacted. Importantly, the pedagogic practices of mobilization and reverberation remind us of the strategic trajectory and political weight of HIC schools in Latin America to advance towards a socially just habitat. For example, FUCVAM’s intergenerational pedagogies are not static practices, but have responded to the Federation’s changing relations to the state over time – building capacities to act in the absence of, against, or with government institutions depending on their (legal) support for cooperative housing models. Finally, the schools have been fertile grounds for learning and acting in solidarity, as witnessed through their provoking of alternative economic, cultural and environmental models and imaginaries of *buen vivir*, as in the case of the *Barrio Intercultural.*

This paper is ultimately a call for humility to those of us engaged in academia and professional practice, and for acknowledgement and recognition of and deep engagement with the actual grounded pedagogies that activate transformative change towards urban equality across time and geographies. In simple terms, it is about activating the practice of collective freedom and, in doing so, creating paradise not just in the classrooms of higher education institutions, but through the multiple sites in which urban inequalities are experienced and effectively contested and counteracted.

## Footnotes

1. hooks (1994), page 207.

2. Allen et al. (2020).

3. Wesely et al. (2021).

4. Habitat International Coalition (2016).

5. See reference 3.

6. By “reflexive”, we mean a process of reflection that is intrinsically linked to action, that is opened to question its values, assumptions and practices, as well as participants’ roles in relation to others.

7. See Pírez (2016); Schmid et al. (2018); Streule et al. (2020).

8. See Allen (2021); Castells (2016); Ortiz Flores (2017); Turner (1976).

9. Castillo-Álvarez (2021); García Alcántar and Quiroz Rothe (2017).

10. Enrique Ortíz Flores was HIC’s Secretary General and President during 1988–1999 and 2003–2007 respectively. He was also advisor and civil servant to the Mexican government in 1982–1988, specifically in the National Fund for Popular Housing (Fonhapo in its Spanish acronym), and co-author of the first national housing programme in Mexico. He is an internationally recognized activist for the right to housing and the defence of the social production of habitat.

11. Abramo (2011).

12. See Yap et al. (2021). The four dimensions explained in this paper are equal distribution, reciprocal recognition, parity political participation and solidarity and mutual care.

13. Freire (2001).

14. Giroux (2019).

15. Gore (1993); hooks (2003).

16. See reference 13.

17. Motta (2017), page 5.

18. See reference 17, page 14.

19. See reference 17, page 2.

20. de Sousa Santos (2018).

21. The pedagogic reflexion with the eight schools in Latin America is part of a wider process open to other member organizations within HIC that seeks to elucidate how learning, mobilization, advocacy and action are simultaneously practised within the Coalition. Since 2021, this process has informed the development of cross-regional co-learning spaces driven by HIC’s members. For more information on this initiative and HIC’s first co-learning space on feminist approaches to habitat rights see: https://www.hic-net.org/how-do-hic-members-and-allies-learn-cross-regionally-to-strengthen-our-collective-advocacy-muscles-on-feminist-approaches-to-habitat/.

22. Enet et al. (2021); Enet and Romero (2019).

23. Gudynas (2011).

24. Malecki (2016).

25. CISCSA et al. (2021).

26. Zaragocin and Caretta (2021), page 1508.

27. Escobar (2020).

28. Rendón (2009), n.p.

29. De Jong et al. (2019), page xv.

30. Illich (1973, 1977, 2010).

31. Corporación Región, Fuerza Incluyente, HIC and KNOW (2021).

32. FUCVAM et al. (2021).

33. See reference 32.

34. As a diverse field, sound and sonic studies are amongst many things interested in the ways sound and acts of listening make differences in society and culture. See for example Gershon (2020).

35. See reference 34, page 1164.

36. Paola Blanes speaking in reference 25.

37. Thompson (1987).

38. Harden (1996), page 32.

39. Luke and Gore (1992).

40. Du Bois (1994).

41. See reference 17, pages 5–6.

42. Caldeira (2017).

43. See reference 13.

44. Motta and Esteves (2014), page 21.
